# Crack Damage Detection Method via Multiple Visual Features and Efficient Multi-Task Learning Model

**DOI:** 10.3390/s18061796

**Published:** 2018-06-02

**Authors:** Baoxian Wang, Weigang Zhao, Po Gao, Yufeng Zhang, Zhe Wang

**Affiliations:** 1 Structure Health Monitoring and Control Institute, Shijiazhuang Tiedao University, Shijiazhuang 050043, China; wangbx@stdu.edu.cn; 2 School of Technology, Beijing Forestry University, Beijing 100083, China; pogao1009@163.com; 3 School of Electrical and Electronic Engineering, Shijiazhuang Tiedao University, Shijiazhuang 050043, China; zhangyf941202@163.com (Y.Z.); ownernew@163.com (Z.W.)

**Keywords:** crack damage detection, multiple visual feature extraction, multi-task learning model, extreme learning machine

## Abstract

This paper proposes an effective and efficient model for concrete crack detection. The presented work consists of two modules: multi-view image feature extraction and multi-task crack region detection. Specifically, multiple visual features (such as texture, edge, etc.) of image regions are calculated, which can suppress various background noises (such as illumination, pockmark, stripe, blurring, etc.). With the computed multiple visual features, a novel crack region detector is advocated using a multi-task learning framework, which involves restraining the variability for different crack region features and emphasizing the separability between crack region features and complex background ones. Furthermore, the extreme learning machine is utilized to construct this multi-task learning model, thereby leading to high computing efficiency and good generalization. Experimental results of the practical concrete images demonstrate that the developed algorithm can achieve favorable crack detection performance compared with traditional crack detectors.

## 1. Introduction

With the rapid development of information technologies, image acquisition systems are used to obtain the surface defect information of concrete structures, and recently, a number of vision-based methods for detecting crack damage have been developed. For the crack regions, their values are generally different from those background contents and can be considered as the separated boundary lines in the image. Therefore, some crack detection methods based on edge analysis are proposed.

Abdelqader et al. conducted an early study on detecting concrete cracks using four edge detection methods [1], which is the prototype of edge-based concrete crack detection. Hutchinson et al. advocated Canny edge detection using a threshold derived from receiver operating characteristics’ analysis [2], but its performance may not be favorable with non-uniform illumination. Albert et al. utilized Sobel and empirical mode decomposition to find cracks [3]. However, only 15 images were utilized in their reported results, and the image spatial resolutions were also not provided. In [4], top-hat transformation was used to detect the local regions with the thresholding operation, but these crack damages may not be detected accurately when the images include complex noises. Cho et al. explored the concrete crack detection model using five different edge detectors, respectively, and compared their detection performances with different photograph distances [5]. The combination of the Prewitt edge detector and the Otsu method was developed in [6] and has achieved some good detection results, which depended largely on the morphological filter for removing the background false alarms. With the rough Canny detection results, K-means clustering technique was exploited to find the accurate crack regions in [7]. Medina et al. further adopted the Gabor filter invariant model for crack edge detection [8]. Kim et al. applied one hybrid image segmentation model to find the crack regions [9]. A common problem of the three methods mentioned above is that the aided strategy may not work well when the incipient edge detection results are not good.

Because of the non-uniform illuminations and various background clutters, the gray values of one same crack change widely, and the corresponding detection results based on edge analysis may be faulty. To address this issue, crack detectors based on the local analysis are presented. Specifically, the collected image is firstly divided into many regions, and the local classifier is used to select the crack candidate regions. Generally, this type of crack detector consists of two successive parts: feature extraction and crack region detection. With the informative image region descriptor and the effective pattern classification, the crack detection based on local analysis performs better than the general edge-based crack detectors.

As for the feature extraction aspect, Oliveira et al. computed the mean and variance features of image regions, and the crack and non-crack features were separated via the one-class classification strategy [10]. Their adopted mean and variance region features were too simple to obtain good detection results in complex backgrounds. Subsequently, Bray et al. further calculated the histogram features of one image region, and the resultant crack detection performances were improved [11]. Considering the specific edge characteristic of cracks, Xu et al. computed the local statistics features (e.g., crack proportion) with image segmentation [12]. The neighborhood information of the crack region under different scales was exploited to construct the feature vectors in [13]. To deal with the non-uniform illumination, the Local Binary Patterns (LBP) descriptor was adopted for the texture characteristic extraction in [14].

Under the condition of fine concrete aggregate, a neat surface and good lighting, the feature extraction methods mentioned above could obtain the discriminative crack features and non-crack ones. However, influenced by weather changes and complex service environments, the limited representation of one single type of feature might not represent the cracks and non-cracks and cannot guarantee satisfactory crack detection results. Recently, the Deep Learning (DL) model has been used in many image applications. Zhang et al. utilized four Convolutional Neural Networks (CNN) for crack region feature extraction [15]. Cha et al. adopted the Rectified Linear unit (ReLu) function in the CNN model, thereby tending to facilitate much faster computations [16]. The DL-based crack feature extraction often makes for better crack detection results than the usual gray-level features. However, it is well known that the DL technique for crack detection needs to iteratively train the multi-layer network parameters, which is time consuming and possibly leads to the over-fitting of the results.

On the other hand, with the obtained image features, the crack region detection followed needs to construct a feature classifier. Technically speaking, the trained feature classifier determines the crack candidates from those background regions. There are already many cases of crack region classification. Based on the Artificial Neural Network (ANN) model, Xu et al. used the Back Propagation (BP) technique to implement the crack region detection [17]. Owing to the slow training performance of the BP model, an improved BP algorithm with varying slopes of the activation function was presented for crack region detection in [18]. The fully-connected neural network with the multi-layer feature learning model was adopted in [15] and [16], which was trained via the stochastic gradient descent method. Support Vector Machine (SVM) is a powerful classification method based on the structure risk minimization principle. Jahanshahi et al. advocated the combination of ANN and SVM for finding the separate hyperplane between the crack and backgrounds [19]. With the calculated wavelet features of the image region, bridge surface crack detection based on the SVM model was proposed in [20]. To detect various crack defects, Chen et al. presented one binary tree network based on the SVM technique [14].

**Figure 1:**
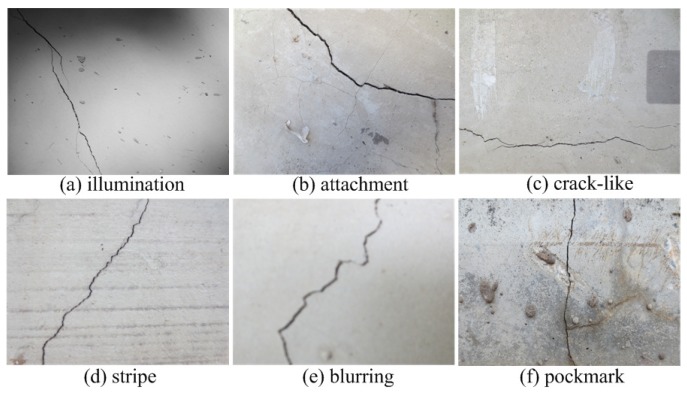
Challenges of concrete crack defect detection in real-world environments.

The aforementioned crack region classification methods have achieved favorable detection performances. However, the ANN-based crack detector needs iterative parameter tuning, and the SVM-based crack detector is faced with a quadratic programming problem. Generally, in order to realize the precise detection of crack defects, one image may be divided into very small regions in actual engineering. The resultant massive image region data will have a high computational burden for these crack detection methods. More importantly, considering the complicated service surroundings of concrete structures, the contents of crack image regions are promiscuous, and the backgrounds contain many disturbances similar to cracks, as shown in Figure 1. As far as we know, most of the existing crack region detection methods simply treat the crack detection task as one binary classification problem [14,15,16,17,18,19,20], which does not fully consider the complexity of image regions, i.e., the variability of crack regions and the disturbance of similar backgrounds.

Through the above analysis, we found that most of the crack detection algorithms based on local analysis cannot achieve optimal performance in terms of accuracy and speed, which can be attributed to the following two aspects. First, the weak feature representation is not appropriate for complex backgrounds, and multi-layer feature extraction is not efficient and is easy to overfit. Second, to deal with massive image region data, the traditional crack region classification is computationally expensive and sensitive to the background clutters.

In this paper, to address the problems above, we attempt to propose a new and effective crack detection model, by exploiting the strong feature learning of multi-view feature extraction and the robustness of multi-task crack region detection. The main contributions are summarized as follows.

1. An efficient feature extraction method is developed for calculating the multi-view image visual features of the crack region, which includes the texture features (i.e., local binary pattern feature) and the edge features (i.e., histogram of oriented gradient feature). By combining these complementary features, the image region’s representation will be enriched and the complex noise disturbances further suppressed.
2. We present a novel crack region detection model based on the multi-task learning framework. Different from the current crack detection approaches, the presented framework not only focuses on the discrimination between cracks and non-cracks, but also fully considers the multiplicity for crack region content. Moreover, an emergent learning technique, i.e., Extreme Learning Machine (ELM), is applied to implement this multi-task framework, thus further improving the efficiency and robustness of the proposed crack detector.
3. The incremental updating equation of the proposed crack region detector has been derived, which makes it very flexible to classify the new crack region candidates with the available up-to-date training image data. Using such an updating equation, the advocated crack detector will be better suited to changing environments.

The remainder of this paper is organized as follows. Section 2 gives an overview of the background content of ELM, which is to implement the developed multi-task classification framework. Section 3 presents the details of the proposed crack detection framework, including the multi-view feature extraction, the multi-task learning classification and the online updating of the crack detector. Experimental results and demonstrations are reported and analyzed in Section 4. Finally, conclusions are given in Section 5.

## 2. Background Content

To facilitate the understanding of the implementation details of the multi-task learning model, we briefly review the theories and concepts of ELM as follows.

The ELM model was originally presented for training the generalized Single hidden Layer Feed-forward Neural network (SLFN) [21] and recently was extended to the multi-layer case [22]. Suppose that one SLFN with *L* hidden nodes can be represented as:

(1) $$f_{L}\left(x\right)=\sum_{i=1}^{L}G\left(w_{i},b_{i},x\right)\gamma_{i}=\sum_{i=1}^{L}h_{i}\left(x\right)\gamma_{i}$$

**Figure 2:**
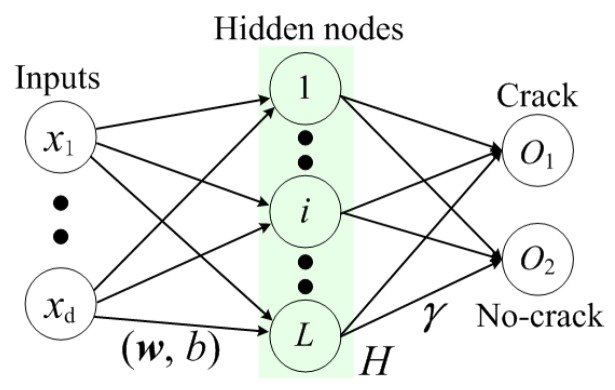
Typical structure of an extreme learning machine framework.

As shown in Equation (1) and Figure 2, $w_{i}$ is the input weight connecting the input *x* to the *i*-th hidden node, and $b_{i}$ is the bias of *i*-th hidden node; G(·) is the activation function; γ is the output weight of the ELM network; $h_{i}\left(\cdot \right)$ is the output vector of the *i*-th hidden node.

Unlike the traditional neural networks, ELM theories show that the hidden neuron parameters can be randomly assigned based on a continuous probability distribution [23]. Specifically, the parameters, i.e., $w_{i}$ and $b_{i}$ of the activation function $G\left(w_{i},b_{i},x\right)$ can be randomly generated without iterative calculation. Therefore, ELM has a much faster learning speed than other learning methods. Moreover, Huang et al. have further proven that the ELM model satisfies the universal classification capability.

Theorem I, classification capability [24]: Given any feature mapping h(x), if h(x)γ is dense in $C(R^{d})$ or in C(M), where *M* is a compact set of $R^{d}$, then SLFN with random hidden layer mapping h(x) can separate arbitrary disjoint regions of any shapes in $R^{d}$ or *M*.

Equation (1) can be rewritten as $f_{L}\left(x\right)=\sum_{i=1}^{L}h_{i}\left(x\right)\gamma_{i}=H\left(x\right)\gamma$. Here, $\gamma ={\left[\gamma_{1},\ldots ,\gamma_{L}\right]}^{T}$ is the matrix of output weights, and $H\left(x\right)=\left[h_{1}\left(x\right),\ldots ,h_{L}\left(x\right)\right]$ is the row vector representing the outputs of *L* hidden nodes. With the randomly generated hidden parameters, H(x) is known to the users. Thus, the ELM function (i.e., Equation (1)) becomes linear, and only the output weights γ are unknown. Given a training dataset $\left\{X,T\right\}={\left\{x^{i},t_{i}\right\}}_{i=1}^{N}$, $x^{i}\in R^{d}$ is the *i*-th training data vector, and $t_{i}\in R^{m}$ represents the corresponding label. The linear equation above can be written in matrix form:

(2) Hγ=T

where H is the hidden layer output matrix (randomized matrix) as follows.

(3) $$H=\left[\begin{array}{c} h(x^{1})\\ \vdots \\ h(x^{N}) \end{array}\right]=\left[\begin{array}{ccc} h_{1}\left(x^{1}\right) & \cdots & h_{L}\left(x^{1}\right)\\ \vdots & \ddots & \vdots \\ h_{1}\left(x^{N}\right) & \cdots & h_{L}\left(x^{N}\right) \end{array}\right]$$

According to the ELM learning algorithm [21], the training of the ELM model is to obtain both the smallest norm of output weights and the smallest training error.

(4) $$\hat{\gamma }=\underset{\gamma }{\arg\min}\left\{{∥\gamma ∥}_{2}^{2}+\lambda {∥T-H\gamma ∥}_{2}^{2}\right\}$$

where λ is a regularization parameter of the training model.

Based on Theorem I mentioned above, recent works have shown that the ELM model achieves good generalization performances in numerous applications, such as human action recognition [25,26], object tracking [27], scene classification [28], hyper-spectral imagery classification [29], etc. Inspired by these, we attempt to apply ELM for efficient and robust crack region detection.

## 3. Proposed Method

**Figure 3:**
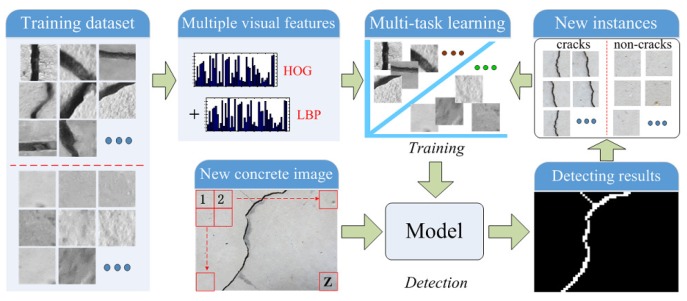
Flowchart of the proposed concrete crack damage detection method.

In this section, we develop a novel crack region detection method, and the overall architecture of the proposed framework is illustrated in Figure 3. One can see that the framework is composed of two stages: (1) training and (2) detection. Before the training stage, by dividing the existing concrete images, many representative crack and non-crack image regions are selected to construct the training dataset. In the training stage, the Histogram of Oriented Gradients (HOG) and LBP features of image regions are firstly calculated. Then, with the computed multi-view features, a novel crack region detection method is advocated using the multi-task learning framework. For one new concrete image, it is divided into many non-overlapping regions, and we apply the trained crack region detector to distinguish these crack candidates from the background ones. With the results of labeling for each testing image region, we perform the morphological image processing as the post-processing to connect discontinuous cracks and remove isolated crack blocks. After the detection stage, some new crack and non-crack training instances are available for incrementally updating the crack detection algorithm.

### 3.1. Multi-View Feature Extraction

**Figure 4:**
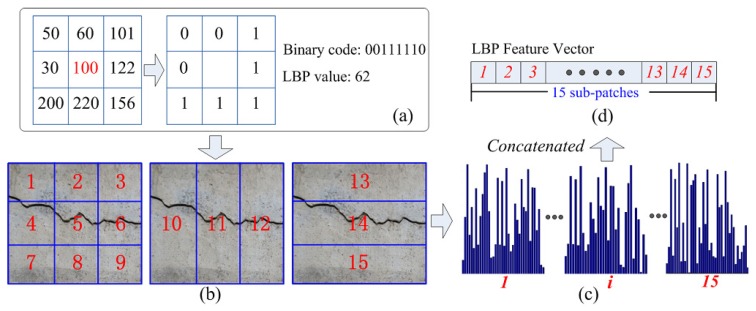
Illustration of LBP feature extraction: (**a**) LBP value generation; (**b**) 15 different sub-patches of the image region; (**c**) LBP features of each sub-patch; (**d**) extracted LBP feature.

**Figure 5:**
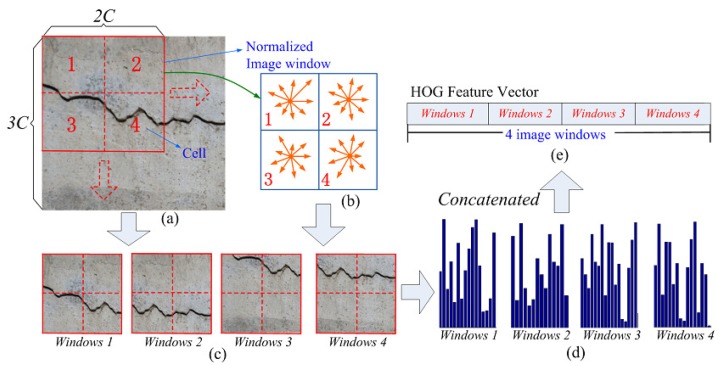
Illustration of HOG feature extraction: (**a**) input image region, (**b**) nine-bin histogram of cell, (**c**) 4 overlapping image windows, (**d**) HOG features of each image window, (**e**) extracted HOG feature.

Due to the limited representation of one single type of feature, most of the current concrete crack detectors may not achieve favorable performances in terms of complex environments. To deal with this representation limitation, we present an efficient scheme that combines two complementary features, i.e., LBP and HOG features of one image region, as shown in Figure 4 and Figure 5.

The LBP model was first advocated by Zabih and Woodfill [30]. For a given pixel *p*, as shown in Figure 4a, the LBP model compares its intensity value with those of its eight neighboring pixels to generate a binary code. By converting the generated binary code into a decimal format, the LBP value of *p* can be obtained, and different LBP values represent different textures around the *p* pixel. It is noted that not all LBP values can represent a meaningful texture, so in this paper, a uniform LBP model [31] is exploited to extract these valid binary codes, which at most have two “1 to 0” or “0 to 1” bit transitions in the binary code. As for computing the LBP features of one predefined image region, LBP values of these pixels in this image region are firstly calculated, and the histogram of all the pixel LBP values is further computed. When computing the histogram, the method accumulates each valid LBP value into a separate bin and keeps all invalid LBP values in a specific bin. Consequently, for one image region, a uniform LBP model will have 58 valid bins and one invalid bin of one histogram. Owing to the fact that the LBP feature captures the texture information of the crack region, which is more robust to illumination changes than other gray-level features, it may not be adaptive to the background clutters. To address this issue, in this work, the position information is exploited by dividing the initial image region into non-overlapping sub-patches. As shown in Figure 4b, for one image region, using different partitioned schemes, there are 15 different sub-patches. By concatenating the histogram entries of each sub-patch, the final LBP feature vector is formed, and its feature dimension is 59 × 15 = 885.

The HOG model computes the histogram of the magnitude sum for gradient orientations in an image region, which is widely used as an effective feature for pedestrian detection [32]. Owing to the crack region having striped characteristics similar to pedestrians, the HOG feature is adopted as the other complementary feature in this paper. Specifically, as shown in Figure 5a, this is implemented by dividing one normalized image window into four small spatial regions named cells. In a cell of C × C pixels, the direction of the gradient at each pixel is discretized into nine bins. Therefore, at each pixel, the gradient is a 2D vector with a real-valued magnitude and a discretized direction (i.e., nine possible directions uniformly distributed in [0, 2π]). Then, the histogram of gradient directions over the pixels of the cell is cumulatively computed, and the calculated nine-bin histogram entries form the representation of each cell. Thus, for one image window, by combining these histogram entries of four cells, we can obtain 36-dimensional region feature vectors. In order to represent more local detail information, the sliding window technique is further utilized, and the sliding step size is C pixels, just as illustrated in Figure 5a. Finally, for an image region of 3C × 3C pixels, it contains four overlapping image windows, and there will be one 144-dimensional (i.e., 144 = 36 × 4) HOG feature vector.

Through the presented image feature extraction mentioned above, the LBP and HOG features of one image region can be easily calculated. By concatenating these feature vectors directly, we can obtain the input sample feature representation of the subsequent ELM-based crack detection model. Compared with the DL-based feature extraction, the proposed multi-view feature extraction does not suffer from the time-consuming feature training process and the risk of over-fitting issues. Moreover, one type of feature captured one piece of channel information of the crack region and compensated for the others’ representation limitation, thereby leading to more robust crack detection results.

### 3.2. Multi-Task Learning Classification

**Figure 6:**
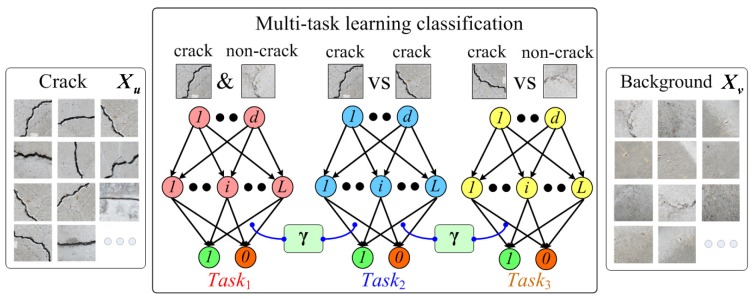
The proposed multi-task learning crack classification method.

As discussed in the previous Section 1, because of the complicated disturbances of the environment, it is difficult to detect the crack regions only considering the discrimination between the cracks and non-cracks; thus, the existing crack detectors based on simple binary classification usually perform poorly. In this section, a multi-task learning classification approach is proposed, just as shown in Figure 6.

Multi-task learning is the procedure of learning several tasks at the same time with the aim of multiple benefits. An early overview of multi-task learning focusing on classification can be found in [33]. Specifically, in this work, the multi-task of the presented crack detector involves three tasks. The first task is used to recognize each single crack or non-crack training sample, which is the basic objective for the crack region detection and is modeled as the first function $f_{task1}\left(x\right)$. The second task is presented to restrain the differences between various crack region features, which can be modeled as the second function $f_{task2}\left(x_{crack1},x_{crack2}\right)$. Unlike the first task that only uses single crack and non-crack samples as the training instances, the crack-crack training pairs are utilized, and the training objective to constrain the consistent outputs of different crack samples would contribute to the crack detection robustness. Finally, the third task is proposed to distinguish the crack candidates from those background noises, which can be modeled as the third function $f_{task3}\left(x_{crack},x_{noncrack}\right)$. Different from the first task, the crack and non-crack training pairs are applied. Therefore, by exploiting the opposite mutual relationship within the training pair, the discrimination between cracks and non-cracks can be further emphasized, thereby leading to more accurate detection performances.

As for the multi-task learning, the three tasks mentioned above should be accomplished within the same framework. Mathematically, we can treat the latter two functions as two different constraints, which are trained with the first function as follows.

(5) $$\left\{\begin{array}{cc} \min & f_{task1}\left(x\right)\\ s.t. & f_{task2}\left(x_{crack1},x_{crack2}\right)\\ s.t. & f_{task3}\left(x_{crack},x_{noncrack}\right) \end{array}\right.$$

Here, $f_{task1}\left(x\right)$ is the basic objective function of the crack region classification task. There exist many approaches for modeling this function $f_{task1}\left(x\right)$, such as SVM, ANN, etc. However, they are generally time-consuming, which hinders their practical use in crack detection. In this paper, we exploit a novel and fast learning technique, namely ELM, to implement this multi-task learning process. Specifically, the solving process of Equation (5) is as follows.

Firstly, as introduced in Section 2, the training of the ELM classification model needs to solve the problem Equation (4), and the output weight γ of the ELM network is the objective to be optimized. Therefore, the first objective function $f_{task1}\left(x\right)$ can be set to be $\left\{{∥\gamma ∥}_{2}^{2}+\lambda {∥T-H\gamma ∥}_{2}^{2}\right\}$. Here, T is the label set of single training samples including crack and non-crack ones, and H=G(w,b,x) is the ELM hidden output of input *x*.

For the latter two constraint equations, two kinds of training pairs are defined as the new training instances. To be specific, one is the crack-crack pair $X_{uu}=\left[x_{crack1},x_{crack2}\right]$, and the other one is the crack-background pair $X_{uv}=\left[x_{crack},x_{noncrack}\right]$. These two sets correspond to the inputs of the latter two constraint equations, just as shown in Equation (5). Technically, for the second task, different crack samples should have approximate outputs of the model. With this rationale, we need to minimize the following problem.

(6) $$\begin{array}{cc} f_{task2}\left(x_{crack1},x_{crack2}\right) & =\min_{\gamma }{∥H(x_{crack1})\gamma -H(x_{crack2})\gamma -M_{uu}∥}_{2}^{2}\\ & =\min_{\gamma }{∥H_{uu}\gamma -M_{uu}∥}_{2}^{2} \end{array}$$

Here, $H(x_{crack})\gamma$ is the output of the ELM classification network for input crack sample $x_{crack}$, and $H(x_{crack})=G\left(w,b,x_{crack}\right)$ is the ELM hidden output of input $x_{crack}$. It should be noted that the randomly generated input hidden parameters $\left(w,b\right)$ are the same as those of the first objective function $f_{task1}\left(x\right)$. For simplicity, the hidden layer output differential value of crack-crack pairs $X_{uu}$ is set to be $H_{uu}$. Moreover, $M_{uu}$ is the label set of the training pairs $X_{uu}$, which indicates the similar relationship between two different crack sample features. To restrain the differences between various crack region features, we set the training labels $M_{uu}$ of crack-crack pairs to zero.

Similarly, for the third task, the crack sample must have a different output from that of the backgrounds, and the following question needs to be solved.

(7) $$\begin{array}{cc} f_{task3}\left(x_{crack},x_{noncrack}\right) & =\min_{\gamma }{∥H(x_{crack})\gamma -H(x_{noncrack})\gamma -M_{uv}∥}_{2}^{2}\\ & =\min_{\gamma }{∥H_{uv}\gamma -M_{uv}∥}_{2}^{2} \end{array}$$

Here, $H_{uv}$ is the hidden layer output differential value of crack-background pairs $X_{uv}$. $M_{uv}$ is the label set of the training pairs $X_{uv}$, which denotes the opposite relationship between the crack and non-crack sample features. To emphasize the discrimination between cracks and non-cracks, the training labels $M_{uv}$ of crack-background pairs are set to one.

It is noteworthy that the three tasks need to be trained in the same ELM network, with the randomly generated input hidden parameters, and the needed ELM output weights γ comprise the only common objective function for all the single and pair training instances. Therefore, for the latter two constraint functions, the smallest norm term of output weights is omitted. The optimization problem of the presented multi-task learning model can be illustrated as follows.

(8) $$\left\{\begin{array}{c} \min_{\gamma }{∥\gamma ∥}_{2}^{2}+\lambda {∥T-H\gamma ∥}_{2}^{2}\\ \;s.t.\;\;{∥M_{uu}-H_{uu}\gamma ∥}_{2}^{2}\\ \;s.t.\;\;{∥M_{uv}-H_{uv}\gamma ∥}_{2}^{2} \end{array}\right.$$

By using the Lagrangian multiplier method, the problem above can be equivalent to one unconstrained optimization problem:

(9) $$\min_{\gamma }\left\{{∥\gamma ∥}_{2}^{2}+\lambda {∥T-H\gamma ∥}_{2}^{2}+\eta {∥M_{uu}-H_{uu}\gamma ∥}_{2}^{2}+\kappa {∥M_{uv}-H_{uv}\gamma ∥}_{2}^{2}\right\}$$

Here, η and κ are the newly-added regularization parameters, which control the penalty weights on the training errors of the latter two learning tasks. The problem Equation (9) is commonly known as ridge regression, and we can easily compute its gradient with respect to γ. By setting the corresponding gradient to zero, we can have the optimal solution as follows.

(10) $$\hat{\gamma }={\left(I+\lambda H^{T}H+\eta H_{uu}^{T}H_{uu}+\kappa H_{uv}^{T}H_{uv}\right)}^{-1}\left(\lambda H^{T}T+\eta H_{uu}^{T}M_{uu}+\kappa H_{uv}^{T}M_{uv}\right)$$

where I is an identity matrix of dimension *L* (i.e., hidden node number of the ELM network). Correspondingly, the final crack region classification decision function is:

(11) $$f\left(x\right)=H\left(x\right)\hat{\gamma }$$

### 3.3. Incremental Model Updating

Considering the continuity of the concrete crack detection task, there will always be new crack and non-crack images in the application of the crack defect detection system. In order to adapt to the changing environments, the presented model has to update the crack detector in a timely manner. An easy way to update the model is to collect all the old and new training instances (including single and pair training samples) for retraining the ELM network. Although this method is easy, using more and more training data is a waste of storage and computation time.

To address this issue mentioned above, in this paper, online sequential updating technology is utilized to update the developed crack region classified network. As for the incremental model updating, the input hidden parameters (i.e., $w_{i}$ and $b_{i}$) are no longer changed. Therefore, we only need to update the output weights γ of the ELM network.

Suppose that we already have $Z_{0}^{\prime }$ single training instances and $Z_{0}^{\prime \prime }$ training pairs including crack-crack and crack-background pairs. Their initial ELM hidden layer outputs are $H_{0}$, $H_{uu0}$ and $H_{uv0}$. The corresponding training labels are $T_{0}$, $M_{uu0}$ and $M_{uv0}$. According to Equation (10), the optimal solution of the initial crack region classification model can be calculated as:

(12) $$\gamma_{0}={\left(I+\lambda H_{0}^{T}H_{0}+\eta H_{uu0}^{T}H_{uu0}+\kappa H_{uv0}^{T}H_{uv0}\right)}^{-1}\left(\lambda H_{0}^{T}T_{0}+\eta H_{uu0}^{T}M_{uu0}+\kappa H_{uv0}^{T}M_{uv0}\right)$$

For simplicity, we rewrite $W_{0}=I+\lambda H_{0}^{T}H_{0}+\eta H_{uu0}^{T}H_{uu0}+\kappa H_{uv0}^{T}H_{uv0}$ and $Q_{0}=\lambda H_{0}^{T}T_{0}+\eta H_{uu0}^{T}M_{uu0}+\kappa H_{uv0}^{T}M_{uv0}$. Then, we can have $\gamma_{0}=W_{0}^{-1}Q_{0}$.

Now, there are $Z_{1}^{\prime }$ new training instances and $Z_{1}^{\prime \prime }$ new training pairs. $T_{1}$, $M_{uu1}$ and $M_{uv1}$ correspond to their training outputs, and we can easily compute their ELM hidden layer output matrix: $H_{1}$, $H_{uu1}$ and $H_{uv1}$. Then, the output weights of the model can be updated as follows.

(13) $$\gamma_{1}=W_{1}^{-1}Q_{1}$$

Considering the old and new sets of training data, we have:

(14) $$\begin{array}{c} \begin{array}{c} W_{1}=I+\lambda {\left[H_{0},H_{1}\right]}^{T}\left[H_{0},H_{1}\right]+\eta {\left[H_{uu0},H_{uu1}\right]}^{T}\left[H_{uu0},H_{uu1}\right]+\kappa {\left[H_{uv0},H_{uv1}\right]}^{T}\left[H_{uv0},H_{uv1}\right]\\ \;\;\;\;\;\;=W_{0}+\lambda H_{1}^{T}H_{1}+\eta H_{uu1}^{T}H_{uu1}+\kappa H_{uv1}^{T}H_{uv1} \end{array} \end{array}$$

(15) $$\begin{array}{c} Q_{1}=\lambda {\left[H_{0},H_{1}\right]}^{T}\left[T_{0},T_{1}\right]+\eta {\left[H_{uu0},H_{uu1}\right]}^{T}\left[M_{uu0},M_{uu1}\right]+\kappa {\left[H_{uv0},H_{uv1}\right]}^{T}\left[M_{uv0},M_{uv1}\right]\\ \;\;\;\;\;=W_{0}\gamma_{0}+\lambda H_{1}^{T}T_{1}+\eta H_{uu1}^{T}M_{uu1}+\kappa H_{uv1}^{T}M_{uv1} \end{array}$$

Substitute Equation (14) into Equation (15), we can have:

(16) $$Q_{1}=W_{1}\gamma_{0}-\lambda H_{1}^{T}H_{1}\gamma_{0}-\eta H_{uu1}^{T}H_{uu1}\gamma_{0}-\kappa H_{uv1}^{T}H_{uv1}\gamma_{0}+\lambda H_{1}^{T}T_{1}+\eta H_{uu1}^{T}M_{uu1}+\kappa H_{uv1}^{T}M_{uv1}$$

Finally, by substituting Equation (16) into Equation (13), we can get the incremental updating equation:

(17) $$\gamma_{1}=\gamma_{0}+W_{1}^{-1}\left(\lambda H_{1}^{T}T_{1}+\eta H_{uu1}^{T}M_{uu1}+\kappa H_{uv1}^{T}M_{uv1}-\lambda H_{1}^{T}H_{1}\gamma_{0}-\eta H_{uu1}^{T}H_{uu1}\gamma_{0}-\kappa H_{uv1}^{T}H_{uv1}\gamma_{0}\right)$$

From the derivations mentioned above, one can see that the presented incremental updating of model could achieve the same learning result as the incipient training with the whole training data including old and new samples. Therefore, it is of good practical significance to carry out the continuous crack defect detection.

## 4. Performance Evaluation and Analysis

### 4.1. Experimental Setup

In this section, to evaluate the proposed crack detection model, we practically collected 350 concrete images by a Canon HS125 camera with a resolution of 4608×3456 pixels. These images contain the typical challenges of concrete crack defect detection in real-world environments, such as illumination, pockmark, stripe, crack-like, attachment, blurring, etc.

The developed crack region detection method is compared with four representative crack detection methods. They are referred to as the Canny-based crack detector [2], the Otsu-based crack detector [6], the SVM-based crack detector [34] and the DL-based crack detector [15]. Specifically, the first two methods are categorized as edge-based crack detectors, and the latter two methods belong to the crack detections based on local analysis. It should be noted that the four compared crack detection methods were implemented by us according to their proposed algorithm framework.

Specifically, for the Canny-based method [2], the built-in edge function of MATLAB is exploited for processing the input concrete images, and the input threshold parameter setting is based on the receiver operating characteristics’ analysis and Bayesian decision theory. As for the Otsu-based method [6], the input images are firstly preprocessed with the Prewitt operator. Then, the built-in function graythresh of MATLAB is applied for segmenting the cracks, and the post morphological processing is further utilized for removing some background noises.

For the SVM-based classified crack detector [34], the mean and variance region features are used as the feature representation of input image samples. The LIBSVM toolbox [35] is adopted to deal with the binary classification problem, and the radial basis kernel function is used in the training and cross-validation processes. The implementation of the DL-based crack detector [15] is based on the MatConvNet [36], which is a MATLAB toolbox implementing CNNs. Specifically, the VGGNet model is used to train the crack detector using many square image regions with the given labels, for the classification of image regions with or without cracks. The entire DL-based crack detection framework consists of four CNNs, four max-pooling and one fully-convolutional network. Here, the filter sizes of the four CNNs are 4 × 4, 5 × 5, 3 × 3 and 4 × 4, respectively. Each CNN is followed by one max-pooling operation, which can learn region features that are spatially invariant. In addition, the fully-connected network with one softmax function is used for crack region classification.

To be fair, all the involved crack detection methods are implemented in the same computing platform (Intel-E5 2.40GHz CPU, GTX960M GPU, 64 GB RAM, Win7 x64 system, MATLAB 2017b). The same training data and testing concrete images are exploited in the local region classification for the compared crack detectors [15,34] and the presented method. To obtain a certain detection ratio of crack defect, in this paper, the size of image regions is set to 75 × 75 pixels. In this section, two popular evaluation criteria are used: one is the Precision Rate (PR), and the other one is the Recall rate (RE), which are defined as follows.

(18) PR=TestTestTTd,RE=TestTestTTg

Here, $T_{est}$ is the number of correctly detected crack regions, $T_{d}$ is the total number of detected crack regions and $T_{g}$ is the number of artificially-labeled crack regions.

### 4.2. Database Generation

The total number of raw concrete images is 350 (4608×3456 pixel resolutions), which were taken from some experimental concrete structures (i.e., beams, deck slab, etc.) at Shijiazhuang Tiedao University. As for the raw images’ collection, we took into account different conditions, e.g., distances, illumination, shadows, blurring, pockmark, and so on. Among the 350 raw concrete images, 250 images were randomly selected for training and validation processes, and the remaining 100 images were for the testing process. As for the training database, the selected images were cropped into small image regions of 75×75 pixel resolution

**Figure 7:**
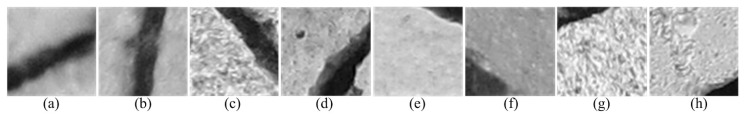
Illustration of crack selection: (**a**–**d**) are the valid instances and (**e**–**h**) are the invalid ones.

As illustrated in Figure 7a–d, for effective crack region detection, the major axis of the crack in one crack sample should be larger than half of the image region size, and the minor axis of the crack should be less than half of the image region size. It is noteworthy that the partitioned images that have cracks on the four corners of image region space are strictly discarded in the training database generation, as shown in Figure 7e–h. To obtain more patterns of cracks or non-cracks, the selected image regions can be rotated by 90 degrees and −90 degrees. Finally, the total number of prepared training image regions is 44K, including 22K crack samples and 22K non-crack samples.

### 4.3. Parameter Setting

In this work, the activation function G(·) is set to be the sigmoid function. In Equation (9), the regularization parameters λ, η, κ are fixed to 0.1, 0.05 and 0.05, respectively, based on the empirical results. As for the second task in Figure 6, we divide the crack dataset $X_{u}$ into two parts and randomly choose 10K cracks from each subset, respectively, which are used to construct 10K crack-crack training pairs $X_{uu}$. Similarly, for the third task in Figure 6, 10K non-crack samples are randomly selected from the background dataset $X_{v}$ and then paired with 10K crack samples for the crack-background training instances $X_{uv}$.

**Figure 8:**
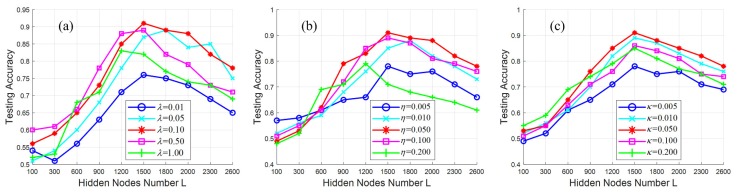
Performances curves of different parameters including *L*, λ, η and κ: (**a**) testing accuracy in terms of *L* and λ, (**b**) testing accuracy in terms of *L* and η, (**c**) testing accuracy in terms of *L* and κ.

The proposed model has some parameters to be tuned, i.e., the hidden neurons number *L* of the ELM-based classification network and the regularization parameters (i.e., λ, η and κ) of Equation (9). Note that *L* represents the Vapnik–Chervonenkis dimension of the ELM classifier. Technically speaking, there is not a best possible way to set the value of *L*. Therefore, it is to be determined by trial-and-error. Figure 8 depicts the testing accuracy curves of different parameter settings. It is noted that the testing accuracy is computed with the testing image regions, which are selected from the 44K training image dataset mentioned above. As shown in Figure 8, when the number *L* is too small, the trained crack detection model has a poor discriminative capability, and it cannot find the cracks from the backgrounds. However, when the number *L* is too large, the resultant crack detector may be too complicated, which makes it difficult to identify the testing image regions, and the testing accuracy begins to decrease. One can see that the proposed crack detection can obtain the optimal results when *L* is 1500.

In addition, the regularization parameters (i.e., λ, η and κ) of Equation (9) control the weights of the three learning tasks. In the experiments, the testing accuracy results with different regularization parameters are also illustrated in Figure 8. From the comparisons, the performance of the presented method can achieve satisfactory results when λ = 0.1, η = κ = 0.05.

### 4.4. Qualitative Evaluation

**Figure 9:**
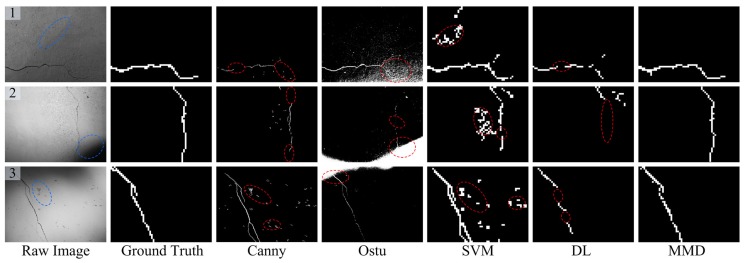
Some representative crack region detection results with illumination changes. DL, Deep Learning; MMD, Multi-view Multi-task crack Detector.

**Figure 10:**
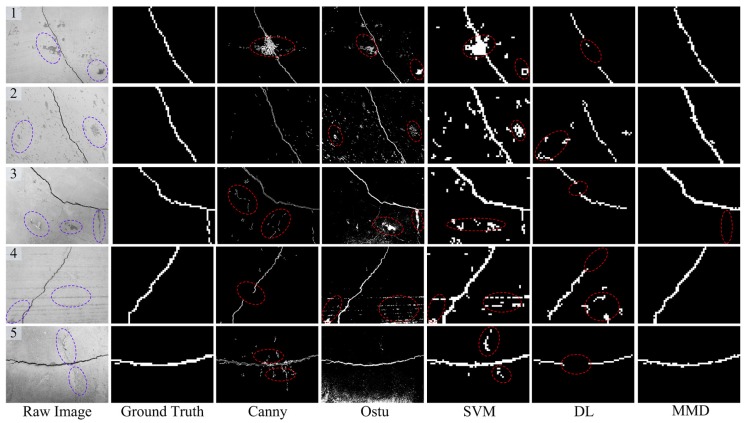
Some representative crack region detection results with background disturbances.

**Figure 11:**
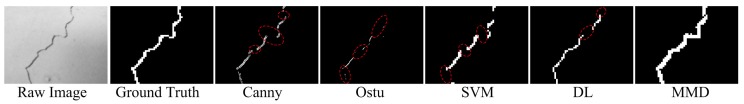
Some representative crack region detection results with image blurring.

**Figure 12:**
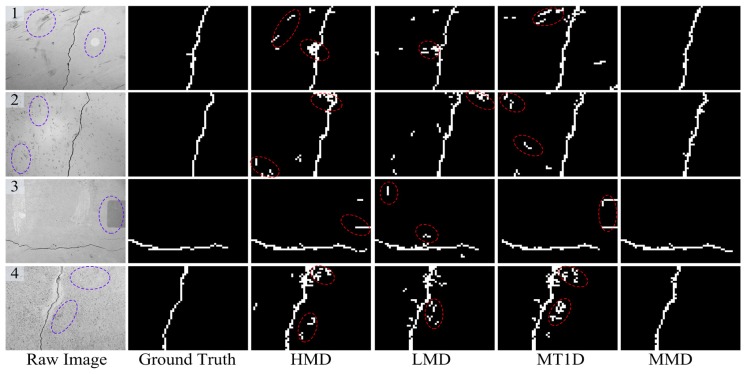
Self-comparisons with three reference crack detection methods. LMD, LBP Multi-task crack Detector; HMD, HOG Multi-task crack Detector; MT1D, Multi-view Task 1 crack Detector.

In this subsection, Figure 9, Figure 10, Figure 11 and Figure 12 show some concrete images that contain illumination changes, background disturbances, crack-like feature, image blurring, etc. Meanwhile, the last five columns of each figure illustrate the crack detection results of Canny [2], Otsu [6], SVM [34], DL [15] and the presented Multi-view Multi-task crack Detector (MMD), respectively. As for the first two compared crack detectors, the concrete images of 4608 × 3456 pixels are processed globally using Canny or Otsu techniques, and the edge-detected or -segmented regions are treated as the crack detection results. On the other hand, for the latter three crack detectors based on local analysis, these concrete images of 4608 × 3456 pixels are firstly divided into 61 × 46 image regions of 75 × 75 pixels. Then, the last three crack detection methods are applied to find the crack regions from those separate candidate ones. In addition, by artificially labeling these divided image regions, the ground truth of concrete images can be obtained, just as shown in the second columns of each figure. It should be noted that the crack region detection results, the size of which is 61 × 46, are enlarged in this illustration for clear comparison. Furthermore, the detailed performance evaluation analyses are as follows.

(1) Illumination changes:

Figure 9 shows some crack detection results for evaluating whether the proposed method is able to tackle illumination changes. For the Canny-based method, in general, the Gaussian filter is used to smooth the background noise. During the image filtering, local tiny cracks may be omitted, just as shown in Figure 9 (2). In addition, the Canny method is sensitive to the background problems (e.g., attachment in Figure 9 (3)), which cannot be removed with simple edge-based techniques. From the detection results of Figure 9, one can see that the Otsu-based method performs worst. The main reason is that there may be several peak values of the gray histogram with non-uniform illuminations. Thus, the dark regions are also segmented and linked together with the true crack regions, which cannot be eliminated via a simple post-processing strategy. By contrast, by using local region binary classification, the SVM-based crack detector can cope with the illumination problems and almost recognize all the crack regions. However, the SVM-based method adopted some simple statistical region features, thereby leading to some false alarms (see the dashed ellipses in Figure 9). Compared with SVM-based method, the DL-based model utilized multi-layer convolutional neural networks for extracting the high-level image region feature, which can well address the background noise (see Figure 9 (1)). However, it may fail to recognize the total crack regions, which may be due to the over-fitting problem.

(2) Background disturbances:

Apart from the illumination issue, there are other disturbances in complex environments, such as pockmarks, attachment, crack-like features, etc. Figure 10 presents the crack region detection results with some background disturbances. With the image filtering technique, the Canny-based method can cope with some tiny background noises, as shown in Figure 10 (2). However, a few blocky background noises are still retained (see Figure 10 (1)). Because of their unknown area and shape parameters, it is difficult to delete them via a simple post-processing operation. Owing to the pixel gray values of certain attachments on the concrete surface being close to that of crack damage, these attachments near the crack region are also segmented (see Figure 10 (1, 3)) by the Otsu-based method. What is more, the shape parameters of these mistakenly segmented areas are various and cannot be removed using post-morphological processing.

As for the SVM-based method, there are also some incorrect detection results like stripes (see Figure 10 (4, 5)), which may be due to the following two reasons. One is that it only utilizes the simple region statistical features, and the other one may be the simple binary classification used in the SVM-based one. For the DL-based crack detector, the pockmark or attachment disturbances can be recognized via the strong feature learning capability, just as shown in Figure 10 (1, 3). On the other hand, it sometimes may be unable to recognize the whole crack regions. For example, from the results of Figure 10 (1, 3, 5), the middle parts of the crack are falsely identified as the backgrounds, which may be due to the over-fitting issue.

From the comparisons mentioned above, it can be seen that the proposed crack detection method has achieved satisfactory detection results because of the following two aspects: (1) the combined complementary image region features (i.e., LBP and HOG) have a strong discriminative capability for dealing with the various background noises; (2) the developed multi-task learning framework contributes to the robustness of the crack region detector when addressing the complex background disturbances. What is noteworthy is that the advocated crack detection method cannot always acquire the perfect crack region detection results. For instance, as illustrated in Figure 10 (3), the lower-right crack regions (see the dashed ellipse) of the input image are not detected. From the point of view of appearance modeling, the visual aspects of undetected tiny crack regions are very similar to those of some crack-like feature (e.g., stripes in Figure 10 (4)). Therefore, to adapt better to complicated surroundings, it is likely that these ambiguous potential crack regions are mistakes.

(3) Image blurring:

There is image blurring or degradation because of the movement during the concrete image capture process, which may cause difficulty in detecting the true crack regions. Generally, image blurring makes the boundary lines of cracks unclear, and thus, these crack detection methods based on edge analysis (i.e., Canny and Otsu) fail to separate the whole crack candidate regions, just as shown in Figure 11. Compared to the SVM-based one, the DL and the proposed MMD method perform better in dealing with the blurry image issue. However, the curved parts of the blurry image are not well detected by the DL-based model. In this work, the MMD method exploits multi-view feature extraction, which can provide more informative region features and contribute to more accurate crack detection results.

### 4.5. Self-Validation

To understand the proposed crack detector better, in this subsection, some reference methods are presented for self-comparisons. The first one keeps the multi-task learning classification, but only uses LBP region features, which is named the LBP Multi-task crack Detector (LMD). Compared to the first one, the second one exploits HOG region features, which is named the HOG Multi-task crack Detector (HMD). The third one only considers the first function $f_{task1}\left(x\right)$ in the multi-task learning framework and keeps other settings unchanged, and thus, we name it the Multi-view Task 1 crack Detector (MT1D).

These methods were implemented over all the testing concrete images, and some representative detection results are shown in Figure 12. From these comparisons, we can see that the proposed crack detector achieves a significant improvement over the LMD and HMD methods. That is because the two complementary features are more robust to the unexpected disturbances like illumination, pockmarks, blurring, crack-like features, and so on. In addition, the developed MMD model performs better than the MT1D method thanks to the multi-task learning technique utilized, which contributes to finding a better separate hyperplane between the various crack contents and the complicated backgrounds.

### 4.6. Quantitative Comparisons

In this subsection, we measure the crack detection accuracy of the proposed method against the other ones using two criteria, i.e., PR and RE. Here, the PR measures the ratio between the correctly detected crack region numbers and the detected crack region ones. Obviously, the large PR value of one crack detector indicates that it has a high confidence coefficient for the detected crack results. Besides, the RE is the ratio between the correctly detected crack region numbers and the labeled crack region ones, which is used to describe the rate of residual undetected crack regions. It should be noted that the PR and RE indexes need a specific number of partitioned image regions, and thus, the Canny and Otsu methods cannot be evaluated in this subsection.

For clear performance comparison, the average PRs and REs for all the testing concrete images are shown in Table 1. From the experimental results, we can see that the DL and the proposed MMD method have a larger average PR value than the SVM-based one. The possible reason is that the DL-based one uses the deep feature learning framework, and the MMD model utilizes the multi-view feature extraction, which can address the likely background disturbances well. However, the DL-based method has a smaller average RE value than the proposed model, which may be attributed to the over-fitting problem.

Furthermore, to verify the performance of the developed incremental updating crack detection method named as the IMMDmodel, it is assumed that the crack detector is incrementally trained with the two partitioned training data. To be specific, one half of 44K training samples is firstly applied to train the initial crack region detector via Equation (12). Then, the other half of the 44K training data is utilized to update the crack detector using Equation (17). In these experiments, the resultant crack detection model is tested with the same testing concrete images, and the average PRs and REs are also shown in Table 1. From the comparisons with the MMD method, one can see that the IMMD model has achieved similar detection performances, which validates the detection accuracy of the incremental updating model. Besides, Table 2 shows the average time of two successive training processes. It is obvious that the IMMD method is more efficient than the MMD model using half of the training data.

### 4.7. Comparison of Training Efficiency

One insight of the proposed method is the application of ELM in the multi-task learning classification. As mentioned above, compared with other traditional learning methods (neural network or SVM), ELM can achieve better generalization performance with much faster learning speed, which contributes to the training efficiency of the MMD crack detector. In this work, owing to the edge-based crack detectors having no need for the training process, we only discuss the crack detection algorithms based on local classification. Specifically, we compared the SVM-based crack detector [34], the DL-based crack detector [15] and the MMD method in terms of the training efficiency aspect.

Table 2 shows the training time of each crack detection in dealing with the same amount of image region data. Moreover, the code implementation software also has an effect on the training efficiency. Although all the compared methods were implemented in MATLAB, there are still some differences in carrying out the specific crack detection, which are listed at the bottom of Table 2.

From the comparisons, it is obvious that the proposed MMD model is the most efficient crack detection method, which is thanks to the ELM’s fast training mechanism. In contrast, the SVM-based one needs an iterative calculation to find the optimal binary decision function. Even though the implementation of the SVM-based method utilizes the fast C-mex function, it is still less efficient in handling the large amount of image region data. Among these methods, the DL-based one is the only crack detector that requires a multi-layer feature pre-training task, thereby making the total training process very slow. To improve the calculation speed, graphics processing unit (GPU) acceleration must be introduced, but this is still the least efficient training model.

## 5. Conclusions

In this paper, a novel concrete crack detection method based on a multi-view and multi-task learning model has been presented. First, multiple visual feature extraction has been developed to compute the texture and edge features of the image region. We have shown that these complementary features can enrich the image region’s representation, thereby facilitating the crack detection performance. Second, we present a new multi-task learning classification framework, which not only emphasizes the discrimination between cracks and non-cracks, but also fully considers restraining the variability for different crack regions. Moreover, the efficient ELM technique is utilized to establish this multi-task classification model, thereby contributing to the training efficiency and robustness of the proposed crack detector. Finally, we have designed the online sequential updating of the crack detector, which could be more suited to changeable environments. Finally, numerous experiments were conducted to compare the proposed crack detection method with other detection methods. Both quantitative and qualitative evaluations further demonstrated the effectiveness and robustness of the proposed method.

## Figures and Tables

**Table 1:** Average Precision Rate (PR) and Recall rate (RE) values. The best results are shown in bold font.

Method	SVM	DL	MMD	IMMD
PR	78.6	83.7	**92.3**	91.9
RE	79.5	76.2	89.7	**89.8**

**Table 2:** Training time and implementation of the crack detection. Training time, seconds.

Method	SVM	DL	MMD	IMMD
Training time	213.2	912	29.1	16.9
Implementation	MATLAB + C	MATLAB + GPU	MATLAB	MATLAB
